# Supplementary material for: A widespread family of WYL-domain transcriptional regulators co-localizes with diverse phage defence systems and islands
Source: Nucleic Acids Res. 2022 May 11;50(9):5191–207. doi: 10.1093/nar/gkac334 (PMC9122601; doi:10.1093/nar/gkac334)
Supplement: gkac334_Supplemental_Files [file gkac334_supplemental_files.zip › Picton et al Supp Material 23MAR22.docx]

# Supplementary Materials

**A widespread family of WYL-domain transcriptional regulators co-localises with diverse phage defence systems and islands**

David M. Picton^a^, Joshua D. Harling-Lee^a,b^, Samuel J. Duffner^a^, Sam C. Went^a^, Richard D. Morgan^c^, Jay C. D. Hinton^d^, Tim R. Blower^a,*^

^a^Department of Biosciences, Durham University, Stockton Road, Durham, DH1 3LE, UK.

^b^The Roslin Institute, Royal (Dick) School of Veterinary Studies, University of Edinburgh, Easter Bush Campus, Edinburgh, EH25 9RG, UK.

^c^New England Biolabs, 240 County Road, Ipswich, MA 01938, USA.

^d^Institute of Infection, Veterinary and Ecological Sciences, University of Liverpool, Liverpool, L69 7ZB, UK.

^*^To whom correspondence may be addressed. Email: [timothy.blower@durham.ac.uk](mailto:timothy.blower@durham.ac.uk), tel: +44(0)1913343923.

Keywords: BREX, WYL-domain, Transcription Factor, Regulator, Phage Defence Island

# Supplementary Figures


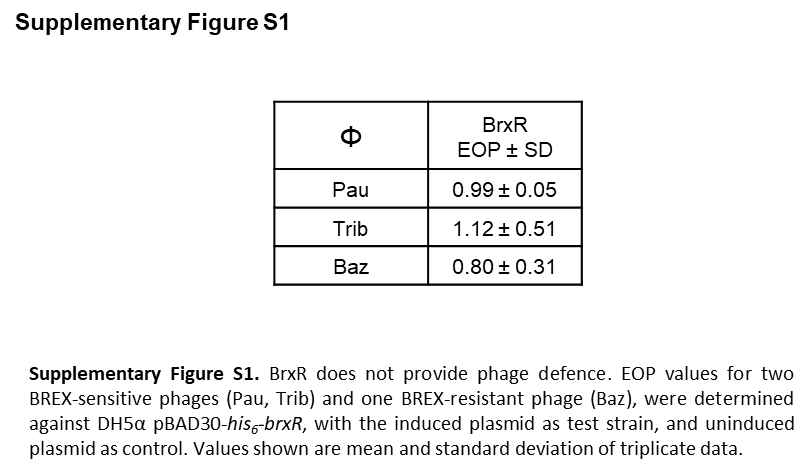


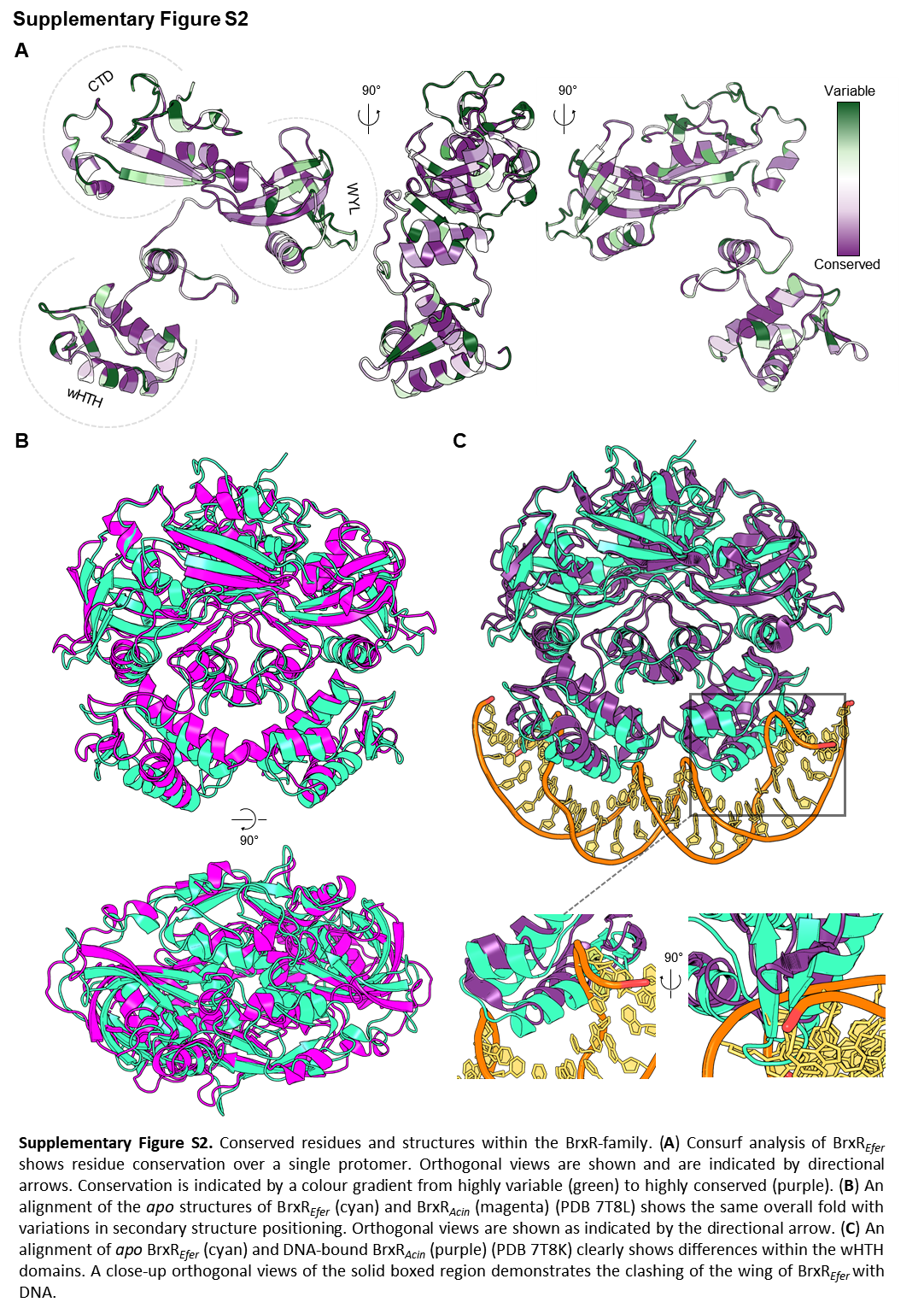


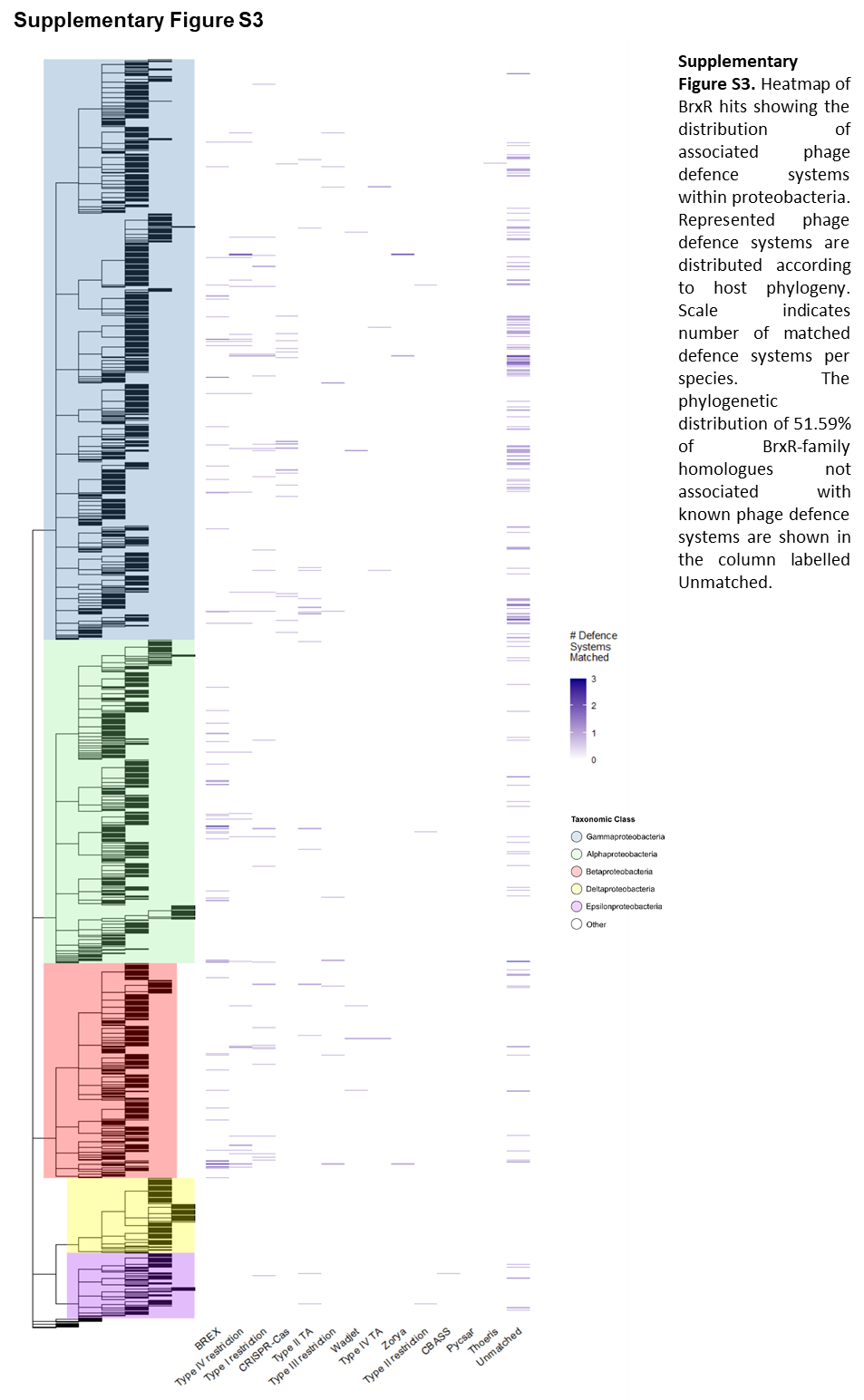


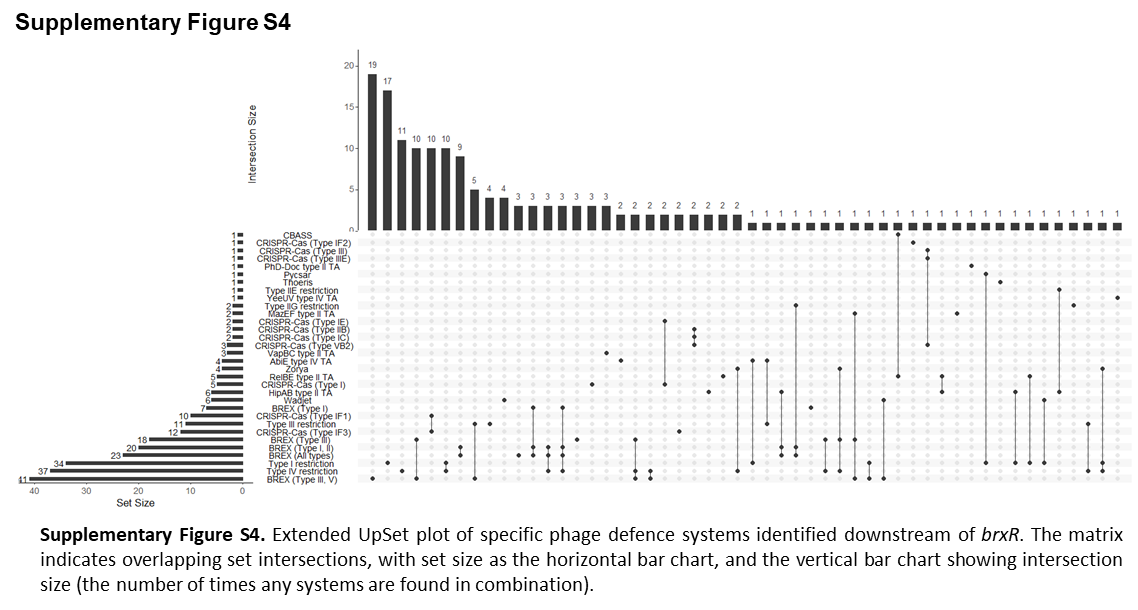


# Supplementary Tables

| **Supplementary Table S1. Oligonucleotides and plasmids used in this study.** | | | | | | |  |  | |  |
| --- | --- | --- | --- | --- | --- | --- | --- | --- | --- | --- |
| **Primer** | **Sequence** | | | **Notes** | | | | | | |
| **pRW50 cloning** | |  | |  | | | | | | |
| TRB904 | TTGAATTCgttatggctggatcacagc | | | FWD pEFER 12400 | | | | | | |
| TRB905 | TTGAATTCcaatggcttgagatggcatg | | | FWD pEFER 13400 | | | | | | |
| TRB906 | TTGAATTCgctgtattgatagactacgc | | | FWD pEFER 13599 | | | | | | |
| TRB907 | TTAAGCTTgccaacggattgttctggc | | | REV pEFER 14007c | | | | | | |
| TRB908 | TTGAATTCggattttactaaacacccgc | | | FWD pEFER 23105 | | | | | | |
| TRB909 | TTGAATTCaaacttggatttcgtcgccg | | | FWD pEFER 23621 | | | | | | |
| TRB910 | TTAAGCTTtgctctgggtcgtaccaaaa | | | REV pEFER 24228c | | | | | | |
| TRB958 | TTAAGCTTaagtccagtgttgtcttgcac | | | REV pEFER 12941c | | | | | | |
| **pBAD30 cloning** | | |  |  | | | | | | |
| TRB876 | TTGAATTCaggagatatcttatgcaccatcaccatcaccatggacaagacaacactggacttgaag | | | FWD RBS, his_6_, pEFER_0020 (*brxR*) | | | | | | |
| TRB877 | TTAAGCTTttattacgattcgctatatccaggagcg | | | REV pEFER *brxR* | | | | | | |
| TRB1987 | agccaagcacagagagaagcactcgctcatattgatttc | | | FWD QuikChange *brxR* R17A | | | | | | |
| TRB1988 | gaaatcaatatgagcgagtgcttctctctgtgcttggct | | | REV QuikChange *brxR* R17A | | | | | | |
| **pSAT1-LIC cloning** | | | |  |  | | | |  |  |
| TRB878 | caacagcagacgggaggtcaagacaacactggacttgaag | | | FWD LIC, pEFER *brxR* | | | | | | |
| TRB879 | gcgagaaccaaggaaaggttattacgattcgctatatccaggagcg | | | REV LIC, pEFER *brxR* | | | | | | |
|  |  | | |  | | | | | | |
| **EMSA probes** | | | |  | | | | | | |
| TRB1067 | TGCGCACTGACAAAAGCTT | | | REV untagged probes | | | | | | |
| TRB1068 | /56-FAM/TGCGCACTGACAAAAGCTT | | | REV fluorescein tagged probes | | | | | | |
| TRB1104 | CAAGTGATTTCTTGAGTTTGAACATTGTTGCGTACAGATATAGTATAGTTTCCGGTGTGAATTCAAGTTCGAAGCTTTTGTCAGTGCGCA | | | Template for P*_rv2827c_* probe | | | | | | |
| TRB1105 | CAAGTGATTTCTTGAGTTTGAACATTG | | | FWD P*_rv2827c_* probe | | | | | | |
| TRB1110 | GACGACTTGTTGATACTATGAAACCTACTGAAAAACAGTAGGTTGCTTGATGGCATTCAATCGATGGCTTAAGCTTTTGTCAGTGCGCA | | | Template for R7 probe | | | | | | |
| TRB1111 | GACGACTTGTTGATACTATGAAACC | | | FWD R7 probe | | | | | | |
| TRB1142 | GACGACTTGTTGATACTATCCCC | | | FWD R7 probe IR1c-IR2 | | | | | | |
| TRB1143 | GACGACTTGTTGATACTATCCCCCCCCCCCAAAAACAGTAGGTTGCTTGATGGCATTCAATCGATGGCTTAAGCTTTTGTCAGTGCGCA | | | Template for R7 IR1c-IR2 probe | | | | | | |
| TRB1144 | GACGACTTGTTGATACTATGAAACCTACTGAAAAACCCCCCCCCCCTTGATGGCATTCAATCGATGGCTTAAGCTTTTGTCAGTGCGCA | | | Template for R7 IR1-IR2c probe | | | | | | |
| TRB1145 | GACGACTTGTTGATACTATCCCCCCCCCCCAAAAACCCCCCCCCCCTTGATGGCATTCAATCGATGGCTTAAGCTTTTGTCAGTGCGCA | | | Template for R7 IR1c-IR2c probe | | | | | | |
|  |  | | |  | | | | | | |
| **Plasmid** | **Notes** | | | **Primers used** | | **Reference** | | | | |
| pRW50 | Tc^R^, promoterless *lacZ* | | | - | | (40) | | | | |
| pRW50-R1 | Tc^R^, pEFER 11001-12399, aka pTRB658 | | | Genscript synthesis | | This study | | | | |
| pRW50-R2 | Tc^R^, pEFER 11001-11378, aka pTRB661 | | | Genscript synthesis | | This study | | | | |
| pRW50-R3 | Tc^R^, pEFER 11379-12399, aka pTRB659 | | | Genscript synthesis | | This study | | | | |
| pRW50-R4 | Tc^R^, pEFER 11980-12399, aka pTRB660 | | | Genscript synthesis | | This study | | | | |
| pRW50-R5 | Tc^R^, pEFER 11980-12109, aka pTRB663 | | | Genscript synthesis | | This study | | | | |
| pRW50-R6 | Tc^R^, pEFER 12400-14007, aka pTRB454 | | | TRB904/907 | | This study | | | | |
| pRW50-R7 | Tc^R^, pEFER 12400-12941, aka pTRB466 | | | TRB904/958 | | This study | | | | |
| pRW50-R7- IR1c-IR2 | Tc^R^, pEFER 12400-12941, polyC in place of IR1 | | | Genscript synthesis | | This study | | | | |
| pRW50-R8 | Tc^R^, pEFER 12682-12941, aka pTRB662 | | | Genscript synthesis | | This study | | | | |
| pRW50-R9 | Tc^R^, pEFER 13400-14007, aka pTRB464 | | | TRB905/907 | | This study | | | | |
| pRW50-R10 | Tc^R^, pEFER 13599-14007, aka pTRB455 | | | TRB906/907 | | This study | | | | |
| pRW50-R11 | Tc^R^, pEFER 23105-24228, aka pTRB452 | | | TRB908/910 | | This study | | | | |
| pRW50-R12 | Tc^R^, pEFER 23621-24228, aka pTRB465 | | | TRB909/910 | | This study | | | | |
| pRW50-P*_abiEi_* | Tc^R^, aka pTRB486 | | | - | | (41) | | | | |
| pRW50-P*_rv2827c_* | Tc^R^, aka pTRB484 | | | - | | (41) | | | | |
| pBAD30 | Ap^R^, D-glu repressed, L-ara induced | | | - | | (39) | | | | |
| pBAD30-*his_6_*-*brxR* | Ap^R^, His_6_-BrxR, aka pTRB451 | | | TRB876/877 | | This study | | | | |
| pBAD30-*his_6_*-*brxR-*R17A | Ap^R^, His_6_-BrxR-R17A | | | TRB1987/1988 | | This study | | | | |
| pSAT1-LIC | Ap^R^, IPTG-inducible | | | - | | (38) | | | | |
| pSAT1-LIC-*brxR* | Ap^R^, His_6_-SUMO-BrxR, aka pTRB441 | | | TRB878/879 | | This study | | | | |

**Supplementary Table S2.** List of DALI hits (excel)

| **Supplementary Table S3. Reference phage defence protein sequences.** | | | |
| --- | --- | --- | --- |
| **Protein** | **Associated with Phage-Defence System** | **Accession** | **Reference** |
| BrxR | NA | WP_017044131.1 | Picton et al., 2021 |
| PglZ | BREX (All types) | WP_004722530.1 | Goldfarb et al., 2015 |
| BrxC | BREX (All types) |  | Goldfarb et al., 2015 |
| Cas3 | CRISPR-Cas (Type I) | WP_037623090.1 | Makarova et al., 2019 |
| Cas9 | CRISPR-Cas (Type II) | WP_024703962.1 | Makarova et al., 2019 |
| Cas10 | CRISPR-Cas (Type III) | WP_014621547.1 | Makarova et al., 2019 |
| PglW | BREX (Type II) | NP_630703.1 - WP_011031052.1 | Goldfarb et al., 2015 |
| BrxP | BREX (Type IV) | YP_001716949.1 - WP_012301903.1 | Goldfarb et al., 2015 |
| BrxHII | BREX (Type III, V) | YP_004121416.1 - WP_013514587.1 | Goldfarb et al., 2015 |
| PglX | BREX (Type I, II) | WP_001095615.1 | Goldfarb et al., 2015 |
| PglXI | BREX (Type III) | YP_004121414.1 – WP_013514585.1 | Goldfarb et al., 2015 |
| BrxU (GmrSD) | Type IV restriction | WP_000283751.1 | Picton et al., 2021 |
| SspC | SspABCD-SspE | WP_016789109.1 | Xiong et al., 2020 |
| DndD | DndABCDE-FGH | WP_042527205.1 | Wang et al., 2007 |
| PvuRts1I | Type IV restriction | WP_011039664.1 | Kazrani et al., 2014 |
| Mrr | Type IV restriction | WP_000217936.1 | Loenen and Raleigh, 2014 |
| McrA | Type IV restriction | WP_000557907.1 | Loenen and Raleigh, 2014 |
| McrB | Type IV restriction | WP_000379041.1 | Loenen and Raleigh, 2014 |
| SauUSI | Type IV restriction | WP_038539189.1 | Loenen and Raleigh, 2014 |
| ScoA3I | Type IV restriction | WP_011030182.1 | Loenen and Raleigh, 2014 |
| MspJI | Type IIM restriction | WP_188871137.1 | Loenen and Raleigh, 2014 |
| DpnI | Type IIM restriction | WP_000418960.1 | Loenen and Raleigh, 2014 |
| MmeI | Type IIL restriction | WP_018986935.1 | Callahan et al., 2016 |
| EcoKI | Type I restriction | WP_001272447.1 | Loenen et al., 2014 |
| EcoprrI | Type I restriction | CAA36526.1 | Loenen et al., 2014 |
| KpnBI | Type I restriction | AAA97402.1 | Loenen et al., 2014 |
| M.StySKI | Type I restriction | EIQ50780.1 | Loenen et al., 2014 |
| Eco57I | Type IIG restriction | WP_032180232.1 | Loenen et al., 2014 |
| BcgI | Type IIB restriction | WP_013853237.1 | Loenen et al., 2014 |
| SapI | Type IIA restriction | TSC79897.1 | Pingoud et al., 2014 |
| BpuSI | Type IIC restriction | WP_098381443.1 | Pingoud et al., 2014 |
| EcoRII | Type IIE restriction | WP_001532073.1 | Pingoud et al., 2014 |
| Cfr10I/Bse634I | Type IIF restriction | WP_072444636.1 | Pingoud et al., 2014 |
| DptF | Type IIH / phosphorothioation-dependent restriction | WP_000417613.1 | Xu et al., 2020 |
| AhdI | Type IIH restriction | WP_168235528.1 | Pingoud et al., 2014 |
| EcoRI | Type IIP restriction | WP_001565219.1 | Pingoud et al., 2014 |
| FokI | Type IIS restriction | AAA24927.1 | Pingoud et al., 2014 |
| BbvCI | Type IIT restriction | AAX14652.1 | Pingoud et al., 2014 |
| EcoP15I | Type III restriction | WP_032190829.1 | Rao et al., 2014 |
| LlaFI | Type III restriction | AAD15793.1 | Rao et al., 2014 |
| PstII | Type III restriction | AAZ73167.1 | Rao et al., 2014 |
| StyLT1 | Type III restriction | AAB26534.1 | Rao et al., 2014 |
| DUF4435 | PARIS | WP_001696664.1 | Rousset et al., 2020 |
| BstA | BstA | WP_000248006.1 | Owen et al., 2021 |
| ZorA | Zorya | WP_186701292.1 | Doron et al., 2018 |
| DrmA | DISARM | WP_227539820.1 | Ofir et al., 2017 |
| pVip8 | Viperins | WP_019672856.1 | Bernheim et al., 2021 |
| NucC | CBASS | WP_001286625.1 | Lau et al., 2020 |
| Cyclase | Pycsar | WP_053265929.1 | Tal et al., 2021 |
| DncV | cGAS | WP_001901330.1 | Cohen et al., 2019 |
| Eco8 | Retron-Eco8 | WP_023304001.1 | Millman et al., 2020 |
| AbiEii | AbiE type IV TA | WP_041980506.1 | Dy et al., 2014 |
| ToxN | ToxIN type III TA | WP_012609144.1 | Fineran et al., 2009 |
| MazF | MazEF type II TA | WP_000254738.1 | Hazan and Engelberg-Kulka, 2004 |
| Hok | Hok/Sok type I TA | WP_001302699.1 | Pecota and Wood, 1996 |
| ThsB | Thoeris | WP_071563695.1 | Doron et al., 2018 |
| JetC | Wadjet | WP_176335405.1 | Doron et al., 2018 |
| Csa5_IA | CRISPR-Cas (Type IA) | WP_010879363.1 | Makarova et al., 2019 |
| Cas8b1_IB | CRISPR-Cas (Type IB) | WP_012103098.1 | Makarova et al., 2019 |
| Cas8c_IC | CRISPR-Cas (Type IC) | WP_010896519.1 | Makarova et al., 2019 |
| Csc3_ID | CRISPR-Cas (Type ID) | ACK70148.1 | Makarova et al., 2019 |
| Cse1_IE | CRISPR-Cas (Type IE) | WP_046891358.1 | Makarova et al., 2019 |
| Csy1_IF1 | CRISPR-Cas (Type IF1) | WP_050296085.1 | Makarova et al., 2019 |
| Cas7f2_IF2 | CRISPR-Cas (Type IF2) | WP_011919225.1 | Makarova et al., 2019 |
| Csy3_IF3 | CRISPR-Cas (Type IF3) | WP_048664206.1 | Makarova et al., 2019 |
| Csb2_IU | CRISPR-Cas (Type IU/IG) | WP_010940732.1 | Makarova et al., 2019 |
| Csf1_IVA | CRISPR-Cas (Type IVA) | ADC73187.1 | Makarova et al., 2019 |
| Csf2_IVB | CRISPR-Cas (Type IVB) | WP_029308491.1 | Makarova et al., 2019 |
| Cas5_IVC | CRISPR-Cas (Type IVC) | RME36479.1 | Makarova et al., 2019 |
| Csm2_IIIA | CRISPR-Cas (Type IIIA) | AAW53329.1 | Makarova et al., 2019 |
| Csm1_IIIA | CRISPR-Cas (Type IIIA) | WP_002486045.1 | Makarova et al., 2019 |
| Cmr1_IIIB | CRISPR-Cas (Type IIIB) | WP_011012270.1 | Makarova et al., 2019 |
| Cmr2_IIIB | CRISPR-Cas (Type IIIB) | WP_011012269.1 | Makarova et al., 2019 |
| Cmr3_IIIB | CRISPR-Cas (Type IIIB) | WP_011012268.1 | Makarova et al., 2019 |
| Cmr4_IIIC | CRISPR-Cas (Type IIIC) | WP_012003242.1 | Makarova et al., 2019 |
| Cas10_IIID | CRISPR-Cas (Type IIID) | WP_011153736.1 | Makarova et al., 2019 |
| Csx10_IIID | CRISPR-Cas (Type IIID) | WP_099887203.1 | Makarova et al., 2019 |
| Cas7_IIIE | CRISPR-Cas (Type IIIE) | KHE91659.1 | Makarova et al., 2019 |
| Csm3_IIIF | CRISPR-Cas (Type IIIF) | WP_012002152.1 | Makarova et al., 2019 |
| Cas9_IIA | CRISPR-Cas (Type IIA) | WP_011227028.1 | Makarova et al., 2019 |
| Cas1_IIB | CRISPR-Cas (Type IIB) | WP_011212793.1 | Makarova et al., 2019 |
| Cas2_IIC1 | CRISPR-Cas (Type IIC1) | WP_002214566.1 | Makarova et al., 2019 |
| Cas2_IIC2 | CRISPR-Cas (Type IIC2) | OJI07265.1 | Makarova et al., 2019 |
| Cpf1_VA | CRISPR-Cas (Type VA) | WP_014550095.1 | Makarova et al., 2019 |
| Cas12b_VB1 | CRISPR-Cas (Type VB1) | WP_021296342.1 | Makarova et al., 2019 |
| Cas4_VB2 | CRISPR-Cas (Type VB2) | QBM02857.1 | Makarova et al., 2019 |
| Cas12c_VC | CRISPR-Cas (Type VC) | KZX85786.1 | Makarova et al., 2019 |
| Cas12d_VD | CRISPR-Cas (Type VD) | OJI08769.1 | Makarova et al., 2019 |
| Cas14a_VF1 | CRISPR-Cas (Type VF1) | QBM01136.1 | Makarova et al., 2019 |
| Cas13a_VIA | CRISPR-Cas (Type VIA) | WP_018451595.1 | Makarova et al., 2019 |
| Cas13d_VID | CRISPR-Cas (Type VID) | WP_215648980.1 | Makarova et al., 2019 |
| Csx28_VIB1 | CRISPR-Cas (Type VIB1) | WP_115154074.1 | Makarova et al., 2019 |
| PglZ_B | BREX Type I | WP_089368483.1 | Goldfarb et al., 2015 |
| EndoR | Type I restriction | WP_153250578.1 | NA |
| VapC | VapBC type II TA | WP_153250579.1 | NA |
| EndoS | Type I restriction | WP_004745828.1 | NA |
| YeeU | YeeUV type IV TA | WP_012154510.1 | Masuda et al., 2012 |
| AbiEii_2 | AbiE type IV TA | WP_012368380.1 | Dy et al., 2014 |
| RelE | RelBE type II TA | WP_014703508.1 | NA |
| EndoM | Type I restriction | WP_015879339.1 | NA |
| VapC_2 | VapBC type II TA | WP_043869448.1 | NA |
| HipA | HipAB type II TA | WP_061904595.1 | NA |
| AbiEi | AbiE type IV TA | WP_081921101.1 | Dy et al., 2014 |
| BrxU_2 | Type IV restriction | WP_089067936.1 | Picton et al., 2021 |
| RelE_2 | RelBE type II TA | WP_095699740.1 | NA |
| BrxF | BREX (Type III) | WP_107219702.1 | Goldfarb et al., 2015 |
| PhD | PhD-Doc type II TA | WP_128384231.1 | NA |
| AbiEii_3 | AbiE type IV TA | WP_142820193.1 | Dy et al., 2014 |
| BrxU_3 | Type IV restriction | WP_178969665.1 | Picton et al., 2021 |
| EndoR_2 | Type I restriction | WP_191600125.1 | NA |
| Cas2_IC | CRISPR-Cas (Type IC) | WP_199275886.1 | Nam et al., 2012 |

**Supplementary Table S4.** List of BrxR hits and associated phage defence systems (excel)

**Supplementary Table S5.** List of additional upstream BrxR-associated phage defence systems (excel)

**Supplementary Table S6.** List of upstream BrxR-associated inverted repeats (excel)
